# Oxidative Stress in Diabetic Retinopathy

**DOI:** 10.3390/antiox10010050

**Published:** 2021-01-04

**Authors:** Ángel L. Ortega

**Affiliations:** Department of Physiology, Faculty of Pharmacy, University of Valencia, Vicente Andrés Estellés Av. s/n, 46100 Burjassot, Spain; angel.ortega@uv.es

Diabetic Retinopathy (DR) is a progressive asymptomatic neuro-vascular complication of diabetes that triggers irreversible retinal damage. This common complication is the leading cause of vision loss in working-age adults (20–65 years) and, consequently, in economically active people [1,2,3]. Although DR is not a life-threatening illness, it leads to emotional distress and reduces daily life functionality, and thus significantly affects the individual’s quality of life [1]. With the worldwide prevalence of diabetes increasing, the number of people with DR is estimated to increase from 424.9 million in 2017 to 628 million by 2045 [4]. This increase in prevalence will make DR one of the main public health burdens.

It is well known that chronic exposure to hyperglycemia induces low-grade inflammation and increases the production of reactive oxygen species with the subsequent loss of redox homeostasis. This contributes to early neuronal retinal cell death [5] and pericytes demise, followed by rupture of the blood retinal barrier, increased vascular permeability [6] and progression to advanced DR stages [6,7,8]. This Special Issue shows DR as a multifactorial disease with a common and complex etiology, including oxidative stress, which calls for a wide range of therapeutic approaches. Miller et al. review the current knowledge on the role of mitochondrial energetic metabolism alteration in the diabetic neural retina and its consequences on retinal function, and suggest the importance of maintaining mitochondrial integrity as a therapeutic strategy [9]. Aragonés et al. summarize the main role of advanced glycation end products (AGEs) in the progression of DR and the potential benefits of enhancing the detoxifying activity of the glyoxalase system as a therapeutic strategy against DR [10]. The harmful role and the involvement of eicosanoids derived from the oxidation of arachidonic acid by the enzymes cyclooxygenase, lipoxygenase, and cytochrome P450 in the development of DR is reviewed by Wang et al. They also propose potential targets and therapies to prevent the development of early-stage DR and progression to proliferative DR [11]. Nebbioso et al. report, as a new therapeutic target, the modulation of the high-mobility group box 1 (HMGB1), a non-histone nuclear protein involved in the inflammatory response and overexpressed under hyperglycemia, contributing to both development and progression to proliferative stages of DR [12].

Current treatments mainly target late-stage DR, when there are already serious vascular alterations and the retina shows neuronal irreparable damages [5]. An earlier diagnosis is therefore key to preventing the ongoing development of DR. López-Contreras et al. highlight the need to study classic and new biomarkers in fluid ocular matrices (tears, aqueous humor, and vitreous), and improve and optimize the sample processing and analysis methods, in order to obtain an early diagnosis and find new therapeutic targets [13]. Adding to new biomarkers and epigenetic modifications, Martins et al. review the little-known role of extracellular vesicles and miRNA in DR development and suggest the potential usefulness of miRNA in combination with anti-inflammatory and/or antioxidant drugs and nutraceutical agents in achieving a personalized therapy [14].

This Special Issue presents nine original research articles showing antioxidant strategies to protect against DR development. The first five manuscripts discuss in vitro approximations. Fernández-Robredo et al. report results showing the antioxidant and anti-inflammatory properties of vitamin D, suggesting its usefulness in moderating the chronic low-grade inflammation and oxidative stress in the development of DR [15]. Oh et al. show the antioxidant capacity of two supplements, ascorbic acid and astaxanthin, using two different oxidative models on a human retinal pigment epithelia cell line (ARPE-19) [16]. Likewise, Lai et al. study the protective pathway induced by astaxanthin against oxidative damage caused by high glucose in mouse photoreceptor cells (661W) [17]. The results show the ability of the carotenoid to activate the PI3K/AKT/Nrf2 pathway and increase the expression of the phase II enzymes NAD(P)H dehydrogenase (NQO1) and heme oxygenase-1 (HO-1), suggesting the use of astaxanthin as a nutritional supplement to prevent visual loss in DR [17]. Hsu et al. study the antioxidant and antiapoptotic properties of a peroxisome proliferator-activated receptor type α (PPAR-α) agonist, fenofibrate, on a monkey choroidal–retinal vascular endothelial cell line (RF/6A) [18]. Fenofibrate enhances thioredoxins 1 and 2 expression and suppresses apoptosis signal-regulated kinase-1 (Ask-1) activity, inhibiting subsequent apoptotic signals [18]. Saenz de Viteri et al. compare different formulations of docosahexaenoic acid and eicosapentaenoic acid supplements mixed in different proportions for the most powerful antioxidant effect on ARPE-19 [19]. Authors suggest that supplements with a higher proportion of eicosapentaenoic acid than docosahexaenoic acid may be more beneficial in preventing or delaying DR progression [19].

Another set of manuscripts presents in vitro experiments combined with in vivo. Kim et al. show the ability of CPA4-1, a herbal combination of *Cinnamomi Ramulus* and *Paeoniae Radix*, to inhibit AGE formation [20]. Moreover, CPA4-1 is able to ameliorate blood-retinal barrier leakage and retinal acellular capillary formation in a mouse model of obesity-induced type 2 diabetes (db/db mice), suggesting CPA4-1 as a potential therapeutic supplement against retinal vascular permeability observed in DR [20]. Ramos et al. examine the possibility of the use of eye drops of glucagon-like peptide-1 (GLP-1) to modulate the antioxidant response in db/db mice. This treatment increases the expression of retinal antioxidant enzymes and prevents DNA/RNA damage, showing neuroprotective activity [21]. Vishwakarma et al. explore the cellular profile and the gene expression related to oxidative stress and pro-inflammatory signaling on the fibrocellular membrane of the eye in three groups of patients: healthy, with proliferative diabetic retinopathy, and with retinal detachment. The analysis shows that oxidative stress and inflammation-associated gene expression increased in patients suffering from proliferative diabetic retinopathy and retinal detachment, providing new information for developing therapies against fibrocellular membrane formation in the late stages of DR [22]. Abouhish et al. show an increase in the expression and activity of histone deacetylase 6 (HDAC6) in human retinal endothelial cells exposed to a high concentration of glucose, in retinas of a rat model of type 1 diabetes, and in human postmortem retinal samples from diabetic patients [23]. Moreover, HDAC6 is related to retinal microvascular hyperpermeability and up-regulation of inflammatory markers, and is presented as a key mediator in hyperglycemia-induced retinal oxidative/nitrative stress in microangiopathy such as DR [23].
